# Timing of coffee consumption and insulin resistance: evidence from human and animal studies

**DOI:** 10.3389/fimmu.2026.1775412

**Published:** 2026-03-13

**Authors:** Peiyan Liu, Guixiang Yao, Yixia Wu, Qi Zhou

**Affiliations:** 1 Department of Clinical Nutrition, Shandong Provincial Hospital Affiliated to Shandong First Medical University, Jinan, Shandong, China; 2 State Key Laboratory for Innovation and Transformation of Luobing Theory, Key Laboratory of Cardiovascular Remodeling and Function Research of MOE, NHC, CAMS and Shandong Province, Department of Cardiology, Qilu Hospital of Shandong University, Jinan, China; 3 Laboratory of Translational Gastroenterology, Department of Gastroenterology, Qilu Hospital of Shandong University, Jinan, Shandong, China

**Keywords:** chrono-nutrition, inflammation, insulin resistance, METS-IR, TG/HDL-C, triglyceride-glucose (TyG) index

## Abstract

**Background:**

The role of coffee consumption timing in insulin resistance (IR), a key driver of cardiovascular-metabolic diseases, remains unclear. This study aimed to investigate the association between the timing of coffee consumption and IR in a large population-based study and animal experiments.

**Methods:**

This study comprised two phases. First, we performed a secondary data analysis of the US National Health and Nutrition Examination Survey (NHANES), involving a cross-sectional sample of 20,460 adults with complete dietary data. Temporal coffee consumption patterns were identified via two-step clustering and associated with insulin resistance indices using multivariable-adjusted regression models. Subsequently, findings were validated experimentally using mouse models.

**Results:**

Two distinct coffee consumption patterns were identified: morning-type (36%) and all-day-type (11%). After multivariable adjustment, only the morning-type pattern was significantly associated with lower IR indices (TyG: β = -0.04; METS-IR: β= -0.59; TG/HDL-C: β= -0.29) and a 17% lower risk of sever IR (OR  =  0.83). Stratified analyses confirmed robustness across coffee intake levels. Notably, coffee consumption timing significantly modified the dose-response relationship between coffee intake amounts and IR indices. Morning-type showed a linear inverse relationship with IR indices, while all-day-type exhibited a J-shaped association. Mediation analysis revealed that inflammatory markers, specifically white blood cell (WBC) count and neutrophil-to-lymphocyte ratio (NLR), partially mediated the beneficial effects of morning coffee consumption on insulin resistance. In mice, morning coffee administration reduced fasting plasma glucose and serum insulin levels, improved glucose tolerance, and lowered proinflammatory cytokines (IL-1β, IL-6, ICAM-1, MCP-1).

**Conclusion:**

Drinking coffee in the morning may be more strongly associated with a lower insulin resistance than drinking coffee later in the day.

## Background

Insulin resistance (IR), characterized by diminished responsiveness and impaired glucose metabolism (1), serve as a key pathophysiological driver of metabolic disorders including type 2 diabetes, atherosclerotic cardiovascular diseases, and metabolic dysfunction-associated steatotic liver disease (2–4). Its global prevalence has risen in parallel with increasing obesity rates (5), underscoring the need for modifiable lifestyle interventions.

Recent advances in chrono-nutrition highlight the critical role of dietary timing in metabolic regulation (6). The circadian system orchestrates rhythmic patterns of insulin secretion and glucose metabolism, with peak insulin sensitivity occurring in the morning and declining progressively throughout the day (7, 8). Circadian disruptions—induced by shift work, sleep irregularities, or mistimed eating—have been shown to exacerbate IR through multiple biological pathways (9).

Coffee, globally consumed beverage rich in bioactive compounds (caffeine, chlorogenic acids, trigonelline) (10), exhibits dual modulatory effects on metabolic health. While epidemiological studies associate coffee consumption with improved insulin sensitivity, reduced inflammation, and lower obesity risk (11, 12), its bioactive components also interact with circadian clock gene expression (13–16). Notably, despite these findings, the association between coffee consumption timing and IR remains poorly understood.

Methodologically, although the hyperinsulinemic-euglycemic clamp (HEC) remains the gold standard for insulin sensitivity assessment (17). Practical limitations necessitate reliable surrogates. Validated indices including the triglyceride-glucose (TyG) index, Metabolic Score for Insulin Resistance (METS-IR), and TG/HDL-C ratio provide clinically feasible alternatives for large-scale studies (18).

Utilizing data from the National Health and Nutrition Examination Survey (NHANES), this study investigated the temporal patterns of coffee consumption in relation to IR assessed through these validated indices. Animal experiments employing mouse models with distinct coffee consumption regimens further substantiated these findings. Therefore, this study aimed to investigate the association between temporal patterns of coffee consumption and insulin resistance in a nationally representative population, and to validate this association using a controlled animal experiment.

## Methods

### Part I: secondary data analysis based on NHANES

#### Data source

Data for this secondary analysis were obtained from the National Health and Nutrition Examination Survey (NHANES) (19). NHANES is an ongoing, continuous, cross-sectional survey conducted by the National Center for Health Statistics (NCHS) of the Centers for Disease Control and Prevention (CDC) to assess the health and nutritional status of the noninstitutionalized civilian population in the United States. It employs a complex, stratified, multistage probability sampling design to ensure national representativeness. The survey protocol was approved by the NCHS Research Ethics Review Board, and all participants provided written informed consent. Further details on ethical approvals are available on the NHANES website (https://www.cdc.gov/nchs/nhanes/irba98.htm).

#### Study population selection

**Figure 1:**
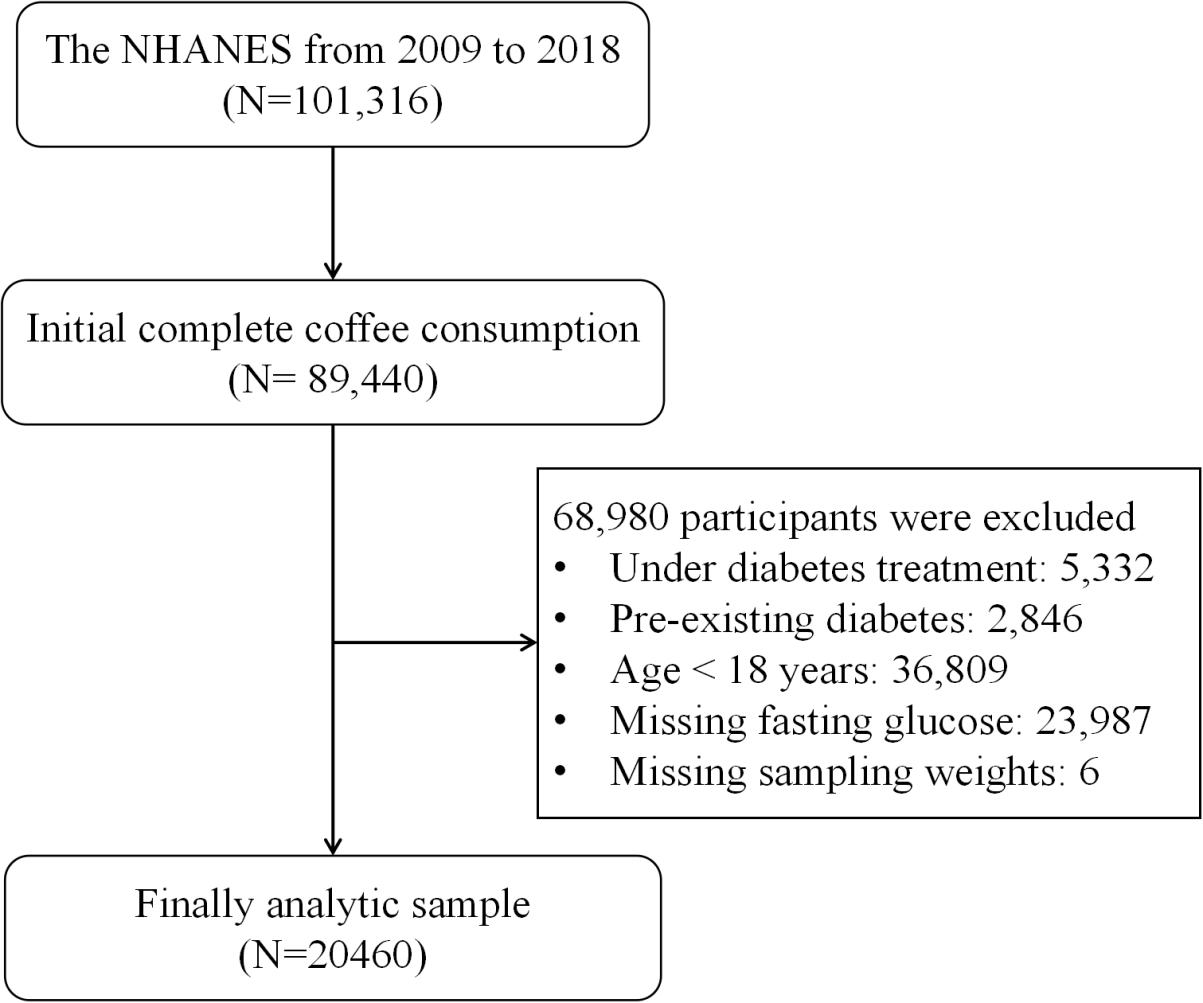
Flowchart of the inclusion and exclusion process. NHANES, National Health and Nutrition Examination Survey.

The analytic cohort for this secondary data analysis was derived from ten consecutive cycles (1999–2018) of the National Health and Nutrition Examination Survey, encompassing 101,316 initial participants. As detailed in **Figure 1**, the study aimed to include adult participants (aged ≥18 years) with complete data on coffee consumption, fasting plasma glucose, and sampling weights. The following exclusion criteria were applied: 1) individuals under diabetes treatment (n=5,332); 2) those with a pre-existing diabetes diagnosis (n=2,846); and 3) those missing data for fasting glucose (n=23,987) or sampling weights (n=6). After applying these criteria to the pool of participants with complete coffee data (N = 89,440), the final analytic sample comprised 20,460 individuals.

#### Assessment of coffee consumption timing patterns

Dietary data were collected using 24-hour dietary recalls, in which participants reported the timing, type, and quantity of all foods and beverages consumed from midnight to midnight on the day preceding the interview. All participants completed an in-person dietary recall during their physical examination. Starting in 2003, a second recall was conducted via telephone 3–10 days later (20). For this study, we primarily analyzed data from the first recall, with sensitivity analyses performed using the second recall.

Coffee intake was quantified by the U.S. Department of Agriculture’s (USDA) Food and Nutrient Database for Dietary Studies (20). The dietary interview questionnaire recorded precise timing of consumption, which we categorized into three periods: morning (4:00 AM–11:59 AM), afternoon (12:00 PM–4:59 PM), and evening (5:00 PM–3:59 AM). The average daily frequency of coffee consumption was calculated for each period, and consumption patterns were identified using cluster analysis.

#### Insulin resistance assessment

The outcome variable was insulin resistance, assessed using three well-established indices: TyG, calculated as ln[fasting triglycerides (mg/dL) × fasting plasma glucose (mg/dL)/2] (21); METS-IR, computed as ln[(2 × fasting plasma glucose (mg/dL) + fasting triglycerides (mg/dL)] × body mass index (kg/m^2^)/ln[HDL-cholesterol (mg/dL)] (22), and the TG/HDL-C, derived by dividing fasting triglyceride levels (mg/dL) by HDL-cholesterol (mg/dL) (23). Higher values in all indices indicate more severe insulin resistance. HOMA-IR was not selected for this analysis due to its reliance on fasting insulin measurements, which are often incomplete in the NHANES dataset and have limited applicability in specific populations (e.g., individuals with abnormal pancreatic function). The alternative methods chosen allow for the maximization of sample size while providing robust indicators of lipid metabolism—a key pathway mediating the physiological benefits associated with coffee consumption.

#### Covariates

This study incorporated multiple covariates based on established evidence from previous research. Continuous variables included age (years), body mass index (BMI), Alternative Healthy Eating Index (HEI) diet score, total energy intake (kcal/day), decaffeinated and caffeinated coffee intake (g/day), and tea consumption (g/day). Categorical variables comprised sex (male/female), race/ethnicity (Non-Hispanic White, Non-Hispanic Black, Mexican American, and other races), family income-to-poverty ratio [low (<1.3)/high (≥1.3)], education level (less than high school/high school or above), marital status (married/unmarried), and hypertension status (yes/no), alcohol consumption (never, former, mild, moderate, and heavy), smoking status (never, former, and current), physical activity level (meeting [≥150 min/week of moderate-intensity activity] or not meeting [<150 min/week] the guideline), and sleep characteristics including duration (<7 hours/≥7 hours per night) and trouble sleeping (yes/no).

### Part II: animal validation

#### Animals and design

6-week-old male C57BL/6J mice were acclimatized for one week and then randomly divided into four groups (n=8): normal chow diet (CON group:10 kcal% fat diet); high-fat diet (HFD, 60% kcal from fat) + saline gavage; HFD+morning coffee gavage (HFD+MC); HFD+all-day coffee gavage (HFD+ADC).

The total daily caffeine dose was fixed at 60 mg/kg/day for both HFD+MC and HFD+ADC groups. This dose is equivalent to moderate human consumption (3–4 cups/day) based on body surface area conversion (24) and aligns with epidemiologically beneficial intake levels (25). The dose was divided into three equal administrations (20 mg/kg each): (1)HFD+MC: Concentrated in a 2-hour window (ZT12, 13, 14) to simulate morning intake; (2)HFD+ADC: Dispersed across the active phase (ZT12, 18, 22) to simulate all-day intake.

#### Solution and procedures

Caffeine solution was prepared daily by dissolving a precise amount of instant coffee (Moccona Classic No.5) in sterile water to achieve target concentration, followed by 0.22-μm filtration. Body weight and food intake were monitored daily. 6-hour fasting blood glucose levels (Accu-Chek Performa, Roche Diagnostics), serum insulin levels (E-EL-M1382, Elabscience), and intraperitoneal glucose tolerance tests (ipGTT; 2 g/kg glucose, tail blood collected at 0, 15, 30, 60, and 120 min) were measured to assess IR. Mouse IL-1β, IL-6, ICAM-1, MCP-1 ELISA kits (E-EL-M0037, E-HSEL-M0005, E-EL-M3037, E-EL-M3001, Elabscience) were used to detect the levels of inflammatory factors in mouse serum.

All protocols were approved by the Institutional Animal Care and Use Committee of Shandong First Medical University and complied with the university’s Guidelines for the Care and Use of Laboratory Animals.

#### Statistical analysis

All analyses incorporated NHANES dietary sampling weights (WTD1YR) to account for the complex, multistage survey design. Continuous variables are presented as weighted means with standard errors (SEs), while categorical variables are reported as counts with corresponding percentages. Group comparisons were performed using Student’s t-tests for continuous variables and chi-square tests for categorical variables for two groups. For comparisons across three or more groups, we used one-way ANOVA with Tukey’s *post hoc* test for continuous variables and chi-square tests for categorical variables. Coffee consumption timing patterns were identified using a two-step clustering approach. A two-step clustering procedure was employed: (1) pre-clustering to generate initial sub-clusters, followed by (2) agglomerative hierarchical clustering. Model selection involved evaluating cluster solutions from 2 to 15, with the optimal number chosen to maximize the average silhouette width. Throughout the process, distances were calculated using log-likelihood, and Schwarz’s Bayesian Criterion guided the clustering. The associations between coffee consumption patterns and insulin resistance indices (TyG, METS-IR, and TG/HDL-C) were evaluated using generalized linear models (GLMs), with β coefficients and 95% confidence intervals (CIs) estimated. Sensitivity analyses were conducted using generalized additive models (GAMs). Three sequential multivariable models were constructed: Model 1 adjusted for age, sex, race/ethnicity, family income, education level, marital status, coffee intake, smoking status, body mass index, and alcohol consumption; Model 2 additionally adjusted for hypertension, sleep duration, and trouble sleeping based on Model 1; and Model 3 (the fully adjusted model) further incorporated healthy eating index, total calorie intake, tea consumption, and physical activity. Additionally, a weighted logistic regression model was used to assess the relationship between severe IR and coffee consumption timing patterns.

Stratified analyses were conducted by age, sex, education, race, sleep duration, trouble sleep, diagnosed hypertension, and physical activity. Additionally, to simultaneously consider both the timing and quantity of coffee consumption, we examined the joint association between temporal patterns of coffee intake and amounts of coffee intake with insulin resistance. Restricted cubic spline (RCS) analysis was used to evaluate the dose-response relationships between coffee consumption levels and three insulin resistance indices (TyG, METS-IR, and TG/HDL-C) across different consumption patterns. Furthermore, we employed mediation analysis to assess whether white blood cell (WBC) count and neutrophil-to-lymphocyte ratio (NLR) mediated the association between coffee consumption timing patterns and insulin resistance markers. The proportion mediated (PM) was calculated to quantify the contribution of WBC count and NLR to the total effect. All analyses were performed using R software (version 4.3.1), with a two-tailed P < 0.05 considered statistically significant.

In the experimental validation section, statistical analyses were performed using one-way analysis of variance (ANOVA) with Tukey’s *post hoc* test for multiple comparisons, as implemented in GraphPad Prism (version 8.0.2). A *p*-value <0.05 was considered statistically significant. In figures, statistical significance is denoted as follows: *p < 0.05, **p < 0.01, and ***p < 0.001, with non-significant results marked as “ns” (not significant).

## Results

### Identification and baseline characterization of coffee consumption temporal patterns

**Figure 2:**
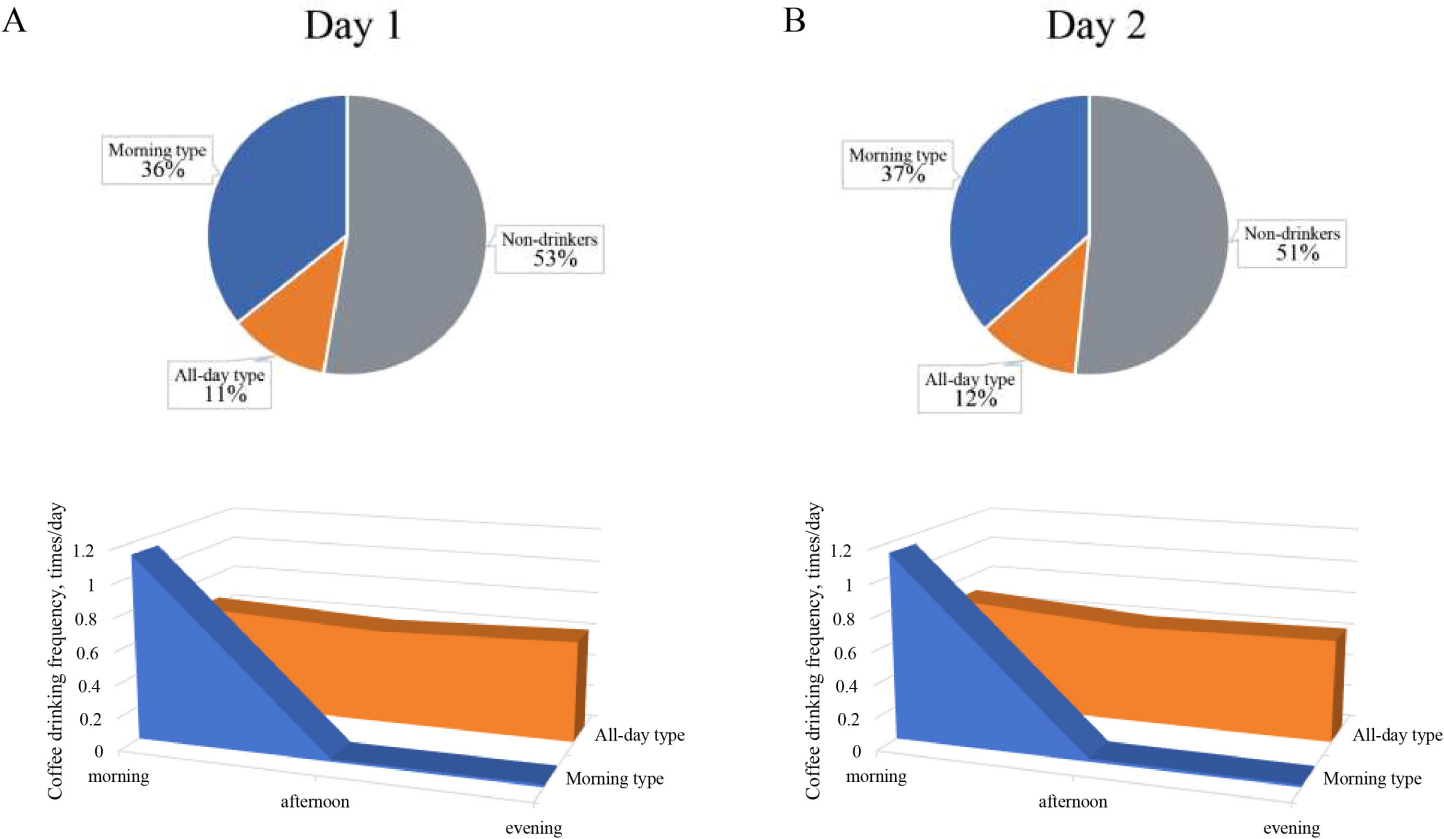
Two-step cluster analysis of coffee consumption temporal patterns in NHANES. Cluster patterns derived from Day 1 **(A)** and Day 2 **(B)** 24-hour dietary recall data. NHANES, National Health and Nutrition Examination Survey.

The study cohort comprised 20,460 participants, of whom 53% (n=10,782) were non-coffee drinkers. Among coffee consumers, cluster analysis identified two distinct temporal consumption patterns: a morning-type pattern (36%, n=7,314) characterized by concentrated coffee intake between 4:00-11:59 AM with minimal afternoon or evening consumption, and an all-day-type pattern (11%, n = 2,364) demonstrating relatively uniform consumption distribution throughout daytime hours (**Figure 2A**). The distinctiveness of these patterns was further illustrated by the heatmap in **Supplementary Figure 1**. Sensitivity analysis using Day 2 dietary data validated these consumption patterns, revealing comparable proportions of morning-type (37%, n = 6,815) and all-day-type consumers (12%, n = 2,184), thus confirming the robustness of the identified temporal consumption patterns (**Figure 2B**).

**Table 1** presents the baseline characteristics. Comparative analysis revealed that both morning-type and all-day-type coffee consumers were significantly older and more likely to be of White ethnicity than non-coffee drinkers. These coffee-consuming groups exhibited higher household income levels, higher rates of hypertension, sleep disorders, and current smoking, but had lower BMI. Among coffee drinkers, morning-type consumers demonstrated distinct behavioral patterns, characterized by greater tea consumption but lower total coffee intake relative to all-day-type consumers. Importantly, morning-type drinkers exhibited significantly lower insulin resistance indices (TyG, METS-IR, and TG/HDL-C) than their all-day-type counterparts.

**Table 1:** Cohort characteristics across coffee consumption time patterns.

Variable	Non-drinkers (N = 10782)	All-day type (N = 2364)	Morning type (N = 7314)	*P*
Age, years	38.67 ± 0.28	50.69 ± 0.48	50.57 ± 0.31	**< 0.001**
Sex, %				0.97
Female	5666(52.01)	1216(51.89)	3800(51.76)	
Male	5116(47.99)	1148(48.11)	3514(48.24)	
Race, %				< 0.001
Non-Hispanic White	4076(62.20)	1170(72.41)	4047(78.28)	
Mexican American	2047(9.12)	442(7.48)	1261(6.27)	
Non-Hispanic Black	2947(15.86)	197(4.42)	913(5.79)	
Other Hispanic	1712(12.82)	555(15.70)	1093(9.66)	
Education levels, %				< 0.001
Less than high school	2861(18.22)	665(18.38)	1858(14.96)	
High school and above	7908(81.78)	1698(81.62)	5448(85.04)	
Marry status, %				< 0.001
Unmarried	4795(44.10)	852(35.05)	2431(31.09)	
Married	5262(55.90)	1458(64.95)	4734(68.91)	
Poverty impact ratio, %				< 0.001
≤1.3	3534(27.49)	661(22.12)	1665(15.92)	
≥1.3	6364(72.51)	1477(77.88)	5054(84.08)	
BMI	28.19 ± 0.11	27.60 ± 0.17	27.83 ± 0.11	**0.005**
Hypertension, %	2975(27.04)	913(35.67)	2978(37.41)	**< 0.001**
Smoke status, %				< 0.001
Never	6133(63.02)	1145(47.35)	3303(44.23)	
Former	1550(16.54)	659(30.00)	2214(31.81)	
Now	1876(20.44)	494(22.65)	1704(23.96)	
Sleep duration, %				0.04
<7 hours	2130(36.35)	428(31.39)	1381(33.54)	
≥7 hours	3443(63.65)	795(68.61)	2369(66.46)	
Trouble sleep, %	1577(23.50)	382(28.69)	1345(28.53)	**<0.001**
Total PA				0.77
<150 min/wk	5350(46.28)	1221(47.11)	3708(45.82)	
≥150 min/wk	5431(53.72)	1143(52.89)	3606(54.18)	
Total calorie intake, kcal	2229.17 ± 13.51	2150.05 ± 29.17	2172.08 ± 14.29	**0.002**
HEl diet score	48.94 ± 0.27	51.85 ± 0.45	51.17 ± 0.28	**< 0.001**
Alcohol consumption				< 0.001
Never	1594(14.94)	265(9.03)	646(6.52)	
Former	1242(11.98)	446(18.85)	1095(13.27)	
Mild	2611(31.13)	816(42.44)	2583(41.19)	
Moderate	1358(16.13)	285(14.93)	1145(19.97)	
Heavy	2167(25.82)	316(14.75)	1290(19.06)	
Tea intake, g	216.76± 7.97	151.09 ± 13.63	170.61± 7.85	**< 0.001**
Total coffee intake, g/day	——	733.19 ± 16.59	593.69 ± 10.88	**< 0.001**
Decaffeinated coffee intake, g/day	——	92.29 ± 8.41	58.83 ± 4.05	**< 0.001**
Caffeinated coffee intake, g/day	——	566.61 ± 17.04	487.07 ± 10.50	**< 0.001**
Index of insulin Resistance				
TyG	8.51 ± 0.01	8.58 ± 0.02	8.56 ± 0.01	**< 0.001**
METS-IR	41.75 ± 0.20	40.63 ± 0.20	40.49 ± 0.29	**< 0.001**
TG/HDL-C	2.84 ± 0.05	2.75 ± 0.08	2.66 ± 0.05	0.06

Data are shown as mean ± SE or n (%). For continuous variables, one−way ANOVA was used for comparisons among three groups, and Student’s t−tests for comparisons between two groups. Categorical variables were compared using chi−square tests.

BMI, body mass index; PA, physical activity; HEI, healthy eating index; TyG, triglyceride-glucose, METS-IR, metabolic score for insulin resistance; TG, triglyceride; HDL-C, high density lipoprotein_cholesterol.

Bold font indicates statistical significance (p < 0.05).

### Association between patterns of coffee drinking timing and insulin resistance indices

Adjusting for age, sex, race/ethnicity, family income, education level, marital status, coffee intake, smoking status, body mass index, and alcohol consumption, the morning coffee consumption pattern showed significant inverse associations with insulin resistance indices compared to non-consumption [TyG: β = -0.06 (95% CI: -0.09, -0.02); METS-IR: β = -0.59 (-0.83, -0.35); TG/HDL-C: β = -0.33 (-0.53, -0.14)]. These associations remained robust after further adjustment for hypertension and sleep disorders [TyG: β = -0.04 (-0.08, -0.01); METS-IR: β = -0.61 (-0.90, -0.32); TG/HDL-C: β = -0.30 (-0.51, -0.08)], and persisted in the fully adjusted model including healthy eating index, total calorie intake, tea consumption, and physical activity [TyG: β = -0.04 (-0.08, 0); METS-IR: β = -0.59 (-0.89, -0.3); TG/HDL-C: β = -0.29 (-0.51, -0.06)]. There was no significant correlation between all-day-type coffee consumption and a lower insulin resistance index (**Table 2**).

**Table 2:** Associations between temporal coffee intake patterns and insulin resistance index.

Model	Non-drinkers	All-day type	Morning type
*TyG*			
Model 1^1^	1 (reference)	-0.03(-0.08, 0.02)	**-0.06(-0.09, -0.02)**
Model 2^2^	1 (reference)	-0.01(-0.07, 0.05)	**-0.04(-0.08, -0.01)**
Model 3^3^	1 (reference)	0(-0.06, 0.06)	**-0.04(-0.08, 0)**
*METS-IR*			
Model 1^1^	1 (reference)	**-0.5(-0.85, -0.14)**	**-0.59(-0.83, -0.35)**
Model 2^2^	1 (reference)	-0.31(-0.75, 0.13)	**-0.61(-0.9, -0.32)**
Model 3^3^	1 (reference)	-0.27(-0.71, 0.18)	**-0.59(-0.89, -0.30)**
*TG/HDL-C*			
Model 1^1^	1 (reference)	-0.21(-0.47, 0.05)	**-0.33(-0.53, -0.14)**
Model 2^2^	1 (reference)	-0.04(-0.35, 0.27)	**-0.3(-0.51, -0.08)**
Model 3^3^	1 (reference)	-0.01(-0.33, 0.30)	**-0.29(-0.51, -0.06)**

Data are presented as beta coefficients (β) and 95% confidence interval (CI). Bold font indicates statistical significance (*p* < 0.05). Analyses were performed using weighted generalized linear models (GLMs).

^1^Model 1 adjusted for age, sex, race and ethnicity, family income, education levels, marry status, coffee intake, smoke status, body mass index and alcohol consumption.

^2^Model 1+ hypertension, short sleep duration, and trouble sleep.

^3^Model 2+healthy eating index, total calorie intake, tea intake, and physical activity.

TyG, triglyceride-glucose; METS-IR, metabolic score for insulin resistance; TG, triglyceride; HDL-C, high density lipoprotein_cholesterol.

In addition, we conducted a sensitivity analysis using the GAMs, which yielded consistent results (**Supplementary Table 1**). A second sensitivity analysis, utilizing Day 2 dietary recall data, similarly confirmed significant inverse associations between morning-type coffee consumption and IR indices (**Supplementary Table 2**). In a third sensitivity analysis, we stratified the sample by TyG index quartiles, with the highest quartile representing severe IR. Morning coffee consumption was associated with an 17% reduced risk of severe IR [OR  =  0.83 (0.72,0.96)] (**Supplementary Table 3**). Sensitivity analyses stratified by coffee type (**Supplementary Table 4**) revealed that morning-type coffee was consistently associated with lower TyG and TG/HDL-C, while all-day-type consumption showed no such benefits. For METS-IR, the protective effect was significant in the no sugar and milk-only subgroups but not in the smaller black coffee subgroup (n=922 for black coffee consumers). Further sensitivity analyses stratified by demographic and lifestyle factors (**Supplementary Table 5**) confirmed that this association held across nearly all subgroups, except Mexican Americans and other races, with a significant graded inverse relationship across coffee consumption patterns (p for trend <0.05).

Mediation analysis revealed that the protective association between morning-type coffee consumption and insulin resistance was partially mediated by inflammatory markers. The proportion mediated by WBC count was 22.7% for TyG, 11.1% for METS-IR, and 13.3% for TG/HDL-C; for NLR, the corresponding proportions were 12.7%, 13.3%, and 13.5%.

### Joint association of coffee intake amounts and patterns of coffee drinking timing with insulin resistance

We conducted stratified analyses by quartiles of coffee consumption to examine the association between temporal coffee consumption patterns and insulin resistance indices across different intake levels. The results demonstrated that morning-type coffee consumption was significantly inversely associated with all insulin resistance indices compared to non-drinkers, with consistent trends across all quartiles. In contrast, all-day-type consumption showed no significant associations with any insulin resistance measures (**Table 3**).

**Table 3:** Association of coffee drinking pattern and insulin resistance index in different coffee consumption levels.

Coffee drinking pattern	Q1 (N = 2571)	Q2 (N = 2278)	Q3 (N = 2591)	Q4 (N = 2238)
*TyG*				
Non-drinkers	1 (reference)	1 (reference)	1 (reference)	1 (reference)
All-day type	0.02(-0.12,0.15)	0.01(0.16,-0.14)	-0.05(-0.16,0.07)	-0.04(-0.14,0.06)
Morning type	**-0.06(-0.11,0.00)**	**-0.07(-0.12,-0.01)**	**-0.04(-0.09,-0.02)**	**-0.08(-0.14,-0.03)**
*METS-IR*				
Non-drinkers	1 (reference)	1 (reference)	1 (reference)	1 (reference)
All-day type	-0.46(-1.33,0.41)	0.26(-0.73,1.25)	-0.71(-1.47,0.05)	-0.04(-0.71,0.63)
Morning type	**-0.66(-1.01,-0.30)**	**-0.4(-0.73,-0.08)**	**-0.64(-1.04,-0.23)**	**-0.71(-1.08,-0.35)**
*TG/HDL-C*				
Non-drinkers	1 (reference)	1 (reference)	1 (reference)	1 (reference)
All-day type	0(-0.63,0.63)	0.35(-0.7,1.39)	-0.36(-0.88,0.15)	-0.12(-0.62,0.38)
Morning type	**-0.39(-0.59,-0.18)**	**-0.34(-0.62,-0.06)**	**-0.3(-0.66,-0.06)**	**-0.48(-0.81,-0.15)**

Data are presented as beta coefficients (β) and 95% confidence interval (CI). Bold font indicates statistical significance (*p* < 0.05). Coffee intake was categorized by quartiles (Q1-Q4). Analyses were performed using weighted generalized linear models (GLMs).

All models were adjusted for age, sex, race and ethnicity, family income, education levels, marry status, smoke status, body mass index, alcohol consumption, hypertension, short sleep duration, trouble sleep, healthy eating index, total calorie intake, tea intake, and physical activity.

TyG, triglyceride-glucose; METS-IR, metabolic score for insulin resistance; TG, triglyceride; HDL-C, high density lipoprotein_cholesterol.

**Figure 3:**
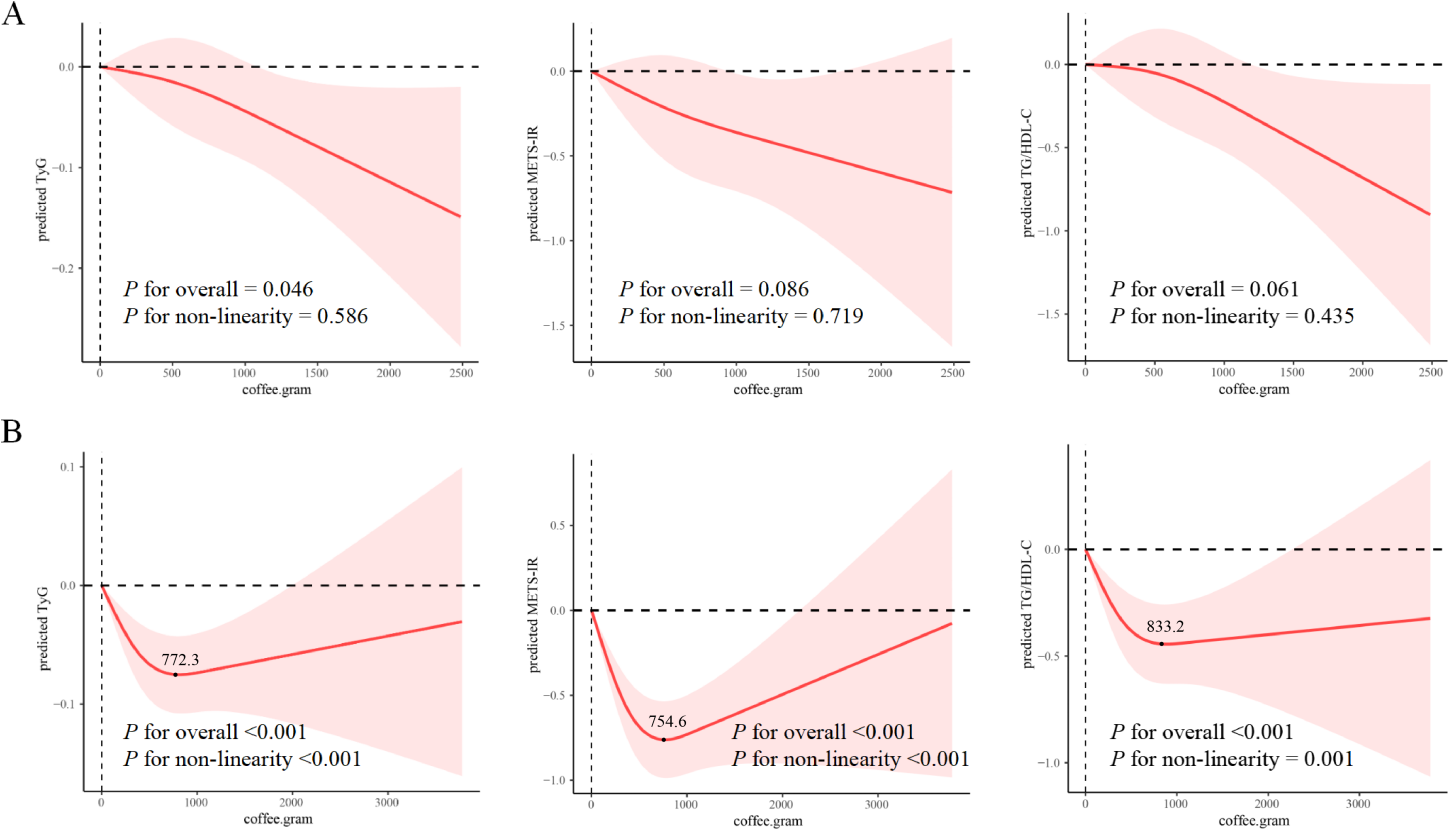
Dose-response association between coffee intake and insulin resistance index by drinking pattern, using restricted cubic spline (RCS) regression. **(A)** Morning type; **(B)** All-day type. Shaded areas represent 95% confidence intervals. *p* for overall tests the significance of the curve; *p* for non-linearity tests departure from linearity. Models were adjusted for all covariates in Model 3 using weighted generalized linear models (GLMs). TyG, triglyceride-glucose; METS-IR, metabolic score for insulin resistance; TG, triglyceride; HDL-C, high-density lipoprotein cholesterol.

Restricted cubic spline (RCS) analyses revealed distinct dose-response patterns: morning-type consumption maintained a consistent linear inverse relationship with insulin resistance indices (P for non-linearity >0.05), while all-day-type consumption followed a J-shaped association (P for non-linearity <0.05) (**Figure 3**). For all-day-type consumption, inflection points were observed at 772.3 g for TyG, 745.6 g for METS-IR, and 833.2 g for TG/HDL-C.

### Drinking coffee in the morning could alleviate IR in mice

**Figure 4:**
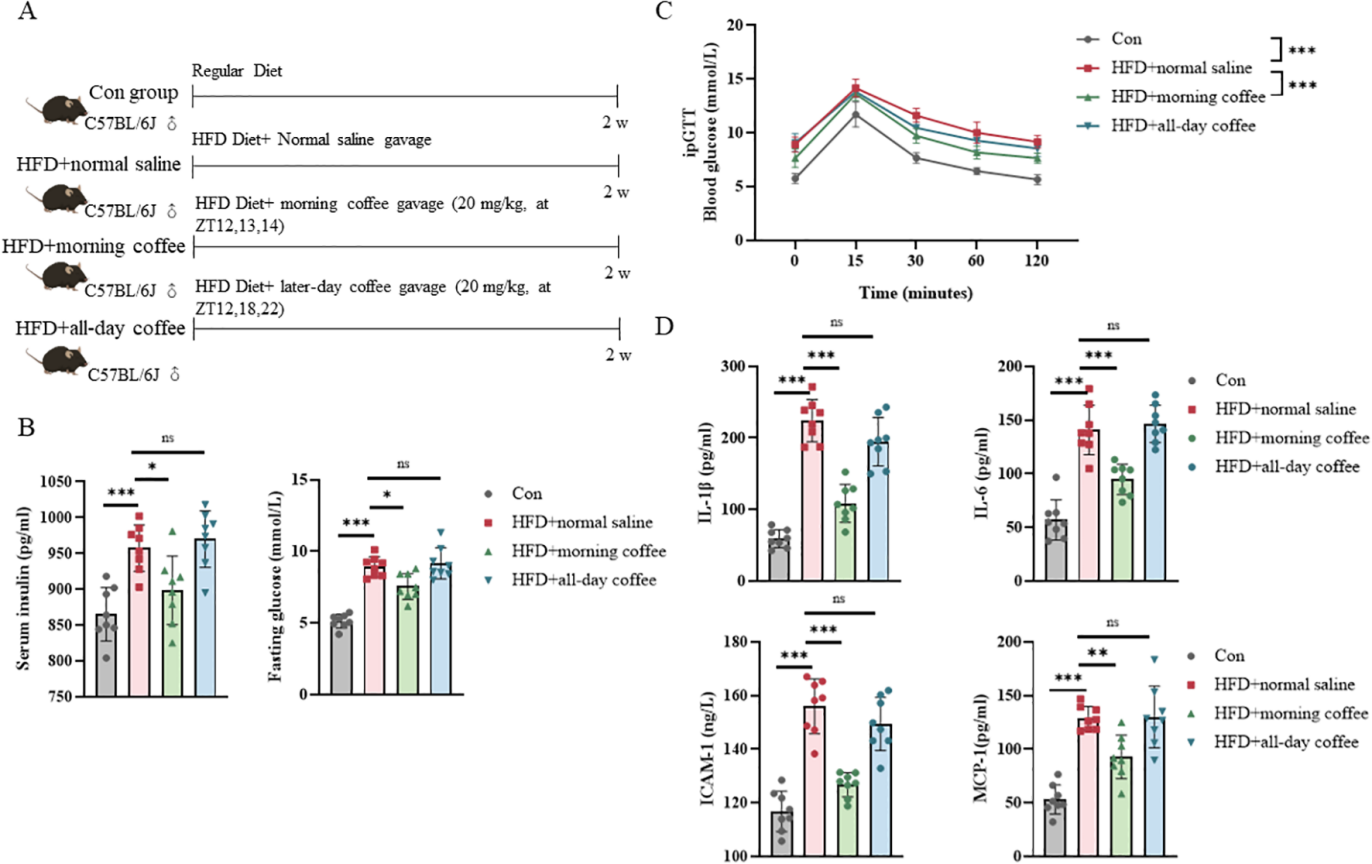
Time-dependent effects of coffee administration on IR in mice. **(A)** Illustration of the experimental study design; **(B)** Serum insulin and fasting blood glucose (6-hour fast) levels; **(C)** Intraperitoneal glucose tolerance test (ipGTT) curves; **(D)** Pro-inflammatory cytokines (IL-1β, IL-6, ICAM-1, and MCP-1) levels. Data are mean ± SEM (n=8/group). Statistical analysis was performed using one-way ANOVA followed by Tukey’s *post-hoc* test *p < 0.05, **p<0.01, and ***p < 0.001.HFD, high-fat diet; IR, insulin resistance.

To investigate the temporal effects of coffee intake on IR, we conducted animal experiments assessing fasting blood glucose, serum insulin levels, and intraperitoneal glucose tolerance tests (ipGTT). The results demonstrated that mice receiving morning-administered coffee exhibited significantly lower fasting plasma glucose and serum insulin levels compared to controls, along with enhanced glucose regulation during ipGTT. In contrast, the all-day coffee consumption group failed to show comparable metabolic improvements. Mechanistically, morning coffee consumption attenuated systemic inflammation, significantly reducing pro-inflammatory cytokines (IL-1β, IL-6, ICAM-1, and MCP-1) (**Figure 4**).

## Discussion

This population-based analysis provides seminal evidence that the temporal pattern of coffee intake influences its association with insulin resistance (IR), a key pathophysiological driver of type 2 diabetes and cardiovascular disease. Analyzing NHANES data from 20,460 participants, we identified two distinct consumption patterns: morning-type (36%) and all-day-type (11%). Morning-type coffee consumption exhibited a robust, linear dose-dependent inverse association with validated IR markers (TyG, METS-IR, and TG/HDL-C), while all-day consumption exhibited a J-shaped association. WBC and NLR partially mediated the beneficial effects of morning coffee consumption. Notably, animal experiments substantiated the benefits of morning coffee consumption.

Our findings might be explained through two potential mechanisms. First, afternoon or evening coffee consumption could disrupt circadian regulation, leading to insulin resistance through multiple biological processes. The hypothalamic suprachiasmatic nucleus (SCN), serving as a central brain clock of the circadian system, misregulates the function of peripheral metabolic organs through dysregulated autonomic nervous system signaling and hormonal pathways (particularly cortisol and melatonin rhythms) (26–29). Delayed morning cortisol peaks or nocturnal cortisol elevation could antagonize melatonin’s glucose-lowering effects while exacerbating hepatic insulin resistance (27, 30). Clinical evidence shows evening coffee consumption reduces nocturnal melatonin secretion by 30%, which may contribute to progressive β-cell dysfunction (31). Circadian misalignment also disrupts the rhythmic expression of metabolic genes in peripheral clocks. In the liver, gluconeogenic enzymes (PEPCK and G6Pase) lose the circadian oscillation (32, 33). In the muscle and adipose tissue, insulin signaling components (GLUT4 and IRS-1) exhibit dysregulation (34–37). These molecular perturbations collectively contribute to systemic insulin resistance.

Second, the timing of coffee intake may influence inflammatory pathways involved in insulin resistance, as supported by growing experimental evidence. White blood cells release various pro-inflammatory mediators, including IL-1β, IL-6, MCP-1, and ICAM-1, which can activate the JNK pathway, induce serine phosphorylation of IRS-1, and subsequently impair PI3K/AKT signaling (38–40), thereby promoting insulin resistance (41–44). Notably, several bioactive compounds in coffee exhibit anti-inflammatory properties. *In vitro* studies have demonstrated that caffeine inhibits the production of key inflammatory mediators such as TNF-α, IL-2, IL-6, and IFN-γ in stimulated lymphocytes and macrophages (45). Animal studies have further shown that caffeine supplementation reduces the mRNA expression of pro-inflammatory cytokines, including IL-1α, IL-1β, and CRP, in the liver and jejunum of mice (46). Mechanistically, caffeine exerts its immunomodulatory effects primarily through antagonism of adenosine A2A receptors and inhibition of phosphodiesterase activity, leading to elevated intracellular cAMP levels and subsequent suppression of pro-inflammatory signaling pathways, including STAT1 phosphorylation (47, 48). Moreover, these inflammatory markers follow endogenous circadian rhythms, peaking in the early morning and reaching their nadir in the late afternoon. Therefore, a morning-concentrated coffee consumption pattern may exert a more pronounced anti-inflammatory effect compared to a dispersed intake pattern throughout the day. While this mechanism is plausible, other pathways may also be involved, and future research is warranted to further elucidate how the timing of coffee intake influences insulin resistance via inflammatory modulation.

Furthermore, our findings indicate that the association between coffee consumption and insulin resistance index is modulated by the temporal pattern of coffee intake: a linear inverse correlation was observed among individuals with a morning-concentrated pattern, whereas a J-shaped association emerged in those with all-day dispersed consumption. Indeed, the relationship between coffee consumption and insulin resistance has been inconsistent. A prior meta-analysis integrating two randomized controlled trials (RCTs) reported no significant effect of coffee consumption on insulin resistance compared to control groups (11), while another meta-analysis demonstrated a significant reduction in the insulin resistance index associated with coffee intake (49). Additionally, a non-RCT study found no significant correlation between coffee consumption and insulin resistance, nor were there notable differences in other glucose metabolism markers (50). The effects of diverse bioactive components in coffee on insulin resistance exhibit notable heterogeneity. Preclinical studies have demonstrated that caffeine exacerbates insulin resistance by competitively blocking adenosine receptors in skeletal muscle, thereby inhibiting glucose uptake and glycogen synthase activity (51, 52). Conversely, a meta-analysis reported no significant impact of coffee consumption on insulin resistance or sensitivity, a discrepancy potentially attributed to chlorogenic acid and other coffee constituents that may counteract the insulin-disruptive effects of caffeine (53, 54). Importantly, consumption timing may modulate these effects through chronobiological mechanisms. Morning intake aligns with endogenous peaks in inflammatory markers, potentially optimizing coffee’s benefits. In contrast, excessive coffee consumption throughout the day, especially in the evening, may lead to caffeine accumulation, antagonize adenosine receptors, suppress nocturnal melatonin secretion, and ultimately impair sleep. These results underscore the importance of considering not only the amount but also the temporal distribution of coffee intake in epidemiological and clinical studies investigating its role in cardiometabolic health.

Insulin resistance is a key driver in the pathogenesis of type 2 diabetes, atherosclerosis, and MASLD. Therefore, mitigating insulin resistance through morning-specific coffee consumption could potentially delay disease progression in at-risk individuals. Integrating this low-cost behavioral strategy into lifestyle interventions may aid in optimizing metabolic health. However, the clinical and public health implications of these findings require validation via long-term randomized controlled trials.

This study has several notable strengths that enhance the validity and significance of our findings. First, to our knowledge, it represents the first investigation to explore the impact of coffee consumption timing on insulin resistance within a nationally representative U.S. sample. Second, the robustness of our findings is substantiated through multiple approaches: (1) the consistent results obtained from three well-validated insulin resistance indices (TyG index, METS-IR, and TG/HDL-C); and (2) the stability of these associations after comprehensive adjustment for potential confounders, including sociodemographic factors, lifestyle behaviors, and dietary patterns; and(3) consistent results from both GLMs and GAMs, with the latter confirming linear dose-response relationships; and(4) integrating inflammation as a mediator reveals the underlying biological mechanisms. Finally, animal studies were conducted to validate the differential regulatory effects of coffee consumption timing on insulin resistance and its impact on pro-inflammatory mediators.

However, this study has some limitations. Firstly, self-reported questionnaires in NHANES, used for exposure and covariate assessment, are prone to recall bias. Secondly, despite controlling for multiple confounders, the observational design leaves room for unmeasured factors that may bias results. Thirdly, the cross-sectional nature of the study precludes establishing a definitive cause-and-effect relationship. Although we supplemented with animal experimental validation, real-world clinical confirmation through randomized controlled trials remains necessary. Fourthly, the findings, based on NHANES data representative of the US population only, cannot be directly applied to other ethnic groups, necessitating validation in diverse cohorts. Finally, although our mediation analysis identified inflammatory markers (WBC and NLR) as potential mediators, the cross-sectional nature of the data precludes establishing temporal causality, and the possibility of reverse causation, whereby insulin resistance itself may promote systemic inflammation, cannot be ruled out.

## Conclusions

Morning coffee consumption showed a significant inverse association with insulin resistance, independent of total daily coffee intake. This beneficial effect might be partially mediated by reduced WBC count and NLR. In contrast, all-day coffee consumption did not exhibit significant protective effects. Animal experiments further confirmed that a morning-specific coffee intake regimen markedly improved insulin sensitivity and attenuated pro-inflammatory cytokine levels. These findings underscore the critical role of consumption timing in modulating the relationship between coffee intake and insulin resistance, suggesting that the temporal pattern of coffee consumption should be considered as a key factor in future investigations of metabolic health.

## Data Availability

The raw data supporting the conclusions of this article will be made available by the authors, without undue reservation.
