# Supplementary material for: Timing of coffee consumption and insulin resistance: evidence from human and animal studies
Source: Front Immunol. 2026 Mar 13;17:1775412. doi: 10.3389/fimmu.2026.1775412 (PMC13021422; doi:10.3389/fimmu.2026.1775412)
Supplement: Supplementary file 1 [file DataSheet1.docx]

**Supplementary Data**

**Figure S1.** Coffee consumption patterns by cluster. Heatmap illustrates distinct temporal distributions: morning-type and all-day-type.

**Table S1.** Association of coffee drinking pattern and insulin resistance index by using the Generalized Additive Model (GAM).

**Table S2.** Association of coffee drinking pattern and insulin resistance index by using the Day 2 dietary data.

**Table S3.** Association of coffee drinking pattern and sever IR.

**Table S4.** Association of coffee drinking pattern with insulin resistance indices, stratified by coffee type.

**Table S5**. **Stratified analyses of** coffee drinking pattern **and METS-IR.**

**
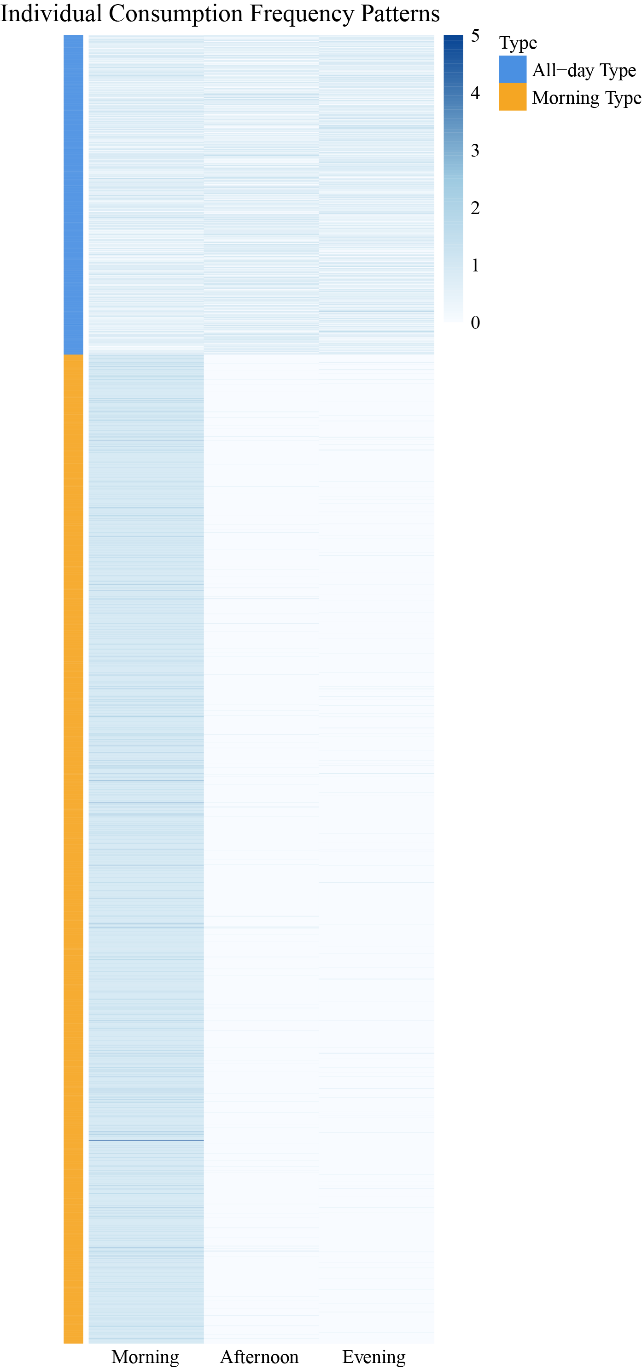
**

**Figure S1.** Coffee consumption patterns by cluster. Heatmap illustrates distinct temporal distributions: morning-type and all-day-type.

**Table S1.** Association of coffee drinking pattern and insulin resistance index by using the Generalized Additive Model (GAM).

| **Model** | **Non-drinkers** | **All-day type** | **Morning type** |
| --- | --- | --- | --- |
| ***TyG*** |  |  |  |
| Full adjust model | 1 (reference) | -0.03(-0.07,0.01) | **-0.07(-0.1,-0.05)** |
| ***METS-IR*** | | | |
| Full adjust model | 1 (reference) | -0.34(-0.62,-0.06) | **-0.62(-0.82,-0.43)** |
| ***TG/HDL-C*** | | | |
| Full adjust model | 1 (reference) | -0.17 (-0.39,0.06) | **-0.41 (-0.56,-0.25)** |

Data are presented as beta coefficients (β) and 95% confidence interval (CI). Bold font indicates statistical significance (*p* < 0.05). Analyses were performed using weighted GAMs.

All models were adjusted for age, sex, race and ethnicity, family income, education levels, marry status, smoke status, body mass index, alcohol consumption, hypertension, short sleep duration, trouble sleep, healthy eating index, total calorie intake, tea intake, and physical activity.

TyG, triglyceride-glucose; METS-IR, metabolic score for insulin resistance; TG, triglyceride; HDL-C, high density lipoprotein_cholesterol.

**Table S2.** Association of coffee drinking pattern and insulin resistance index by using the Day 2 dietary data.

| **Model** | **Non-drinkers** | **All-day type** | **Morning type** |
| --- | --- | --- | --- |
| ***TyG*** |  |  |  |
| Model 1^1^ | 1 (reference) | -0.03(-0.08, 0.02) | **-0.05(-0.08, -0.01)** |
| Model 2^2^ | 1 (reference) | -0.03(-0.08, 0.02) | **-0.06(-0.1, -0.02)** |
| Model 3^3^ | 1 (reference) | -0.03(-0.08, 0.03) | **-0.05(-0.09, -0.01)** |
| ***METS-IR*** | | | |
| Model 1^1^ | 1 (reference) | **-0.52(-0.18, -0.86)** | **-0.59(-0.82, -0.36)** |
| Model 2^2^ | 1 (reference) | -0.29(-0.7, 0.11) | **-0.51(-0.82, -0.2)** |
| Model 3^3^ | 1 (reference) | -0.24(-0.65, 0.18) | **-0.47(-0.78, -0.16)** |
| ***TG/HDL-C*** | | | |
| Model 1^1^ | 1 (reference) | -0.22(-0.48, 0.04) | **-0.33(-0.53, -0.12)** |
| Model 2^2^ | 1 (reference) | -0.01(-0.33, 0.33) | **-0.25(-0.47, -0.04)** |
| Model 3^3^ | 1 (reference) | 0.02(-0.29, 0.33) | **-0.23( -0.44, -0.02)** |

Data are presented as beta coefficients (β) and 95% confidence interval (CI). Bold font indicates statistical significance (*p* < 0.05). Analyses were performed using weighted generalized linear models (GLMs).

^1^Model 1 adjusted for age, sex, race and ethnicity, family income, education levels, marry status, coffee intake, smoke status, body mass index and alcohol consumption.

^2^Model 1+ hypertension, short sleep duration, and trouble sleep.

^3^Model 2+healthy eating index, total calorie intake, tea intake, and physical activity.

TyG, triglyceride-glucose; METS-IR, metabolic score for insulin resistance; TG, triglyceride; HDL-C, high density lipoprotein_cholesterol.

**Table S3.** Association of coffee drinking pattern and sever IR.

| **Model** | **Non-drinkers** | **All-day type** | **Morning type** |
| --- | --- | --- | --- |
| ***TyG*** |  |  |  |
| Model 1^1^ | 1 (reference) | 0.90(0.73,1.11) | **0.81(0.70,0.93)** |
| Model 2^2^ | 1 (reference) | 0.91(0.74,1.12) | **0.82(0.71,0.95)** |
| Model 3^3^ | 1 (reference) | 0.92(0.75,1.13) | **0.83(0.72,0.96)** |

Data are presented as odds ratio (OR) and 95% confidence interval (CI). Bold font indicates statistical significance (*p* < 0.05). Analyses were performed using weighted logistic regression models.

^1^Model 1 adjusted for age, sex, race and ethnicity, family income, education levels, marry status, coffee intake, smoke status, body mass index and alcohol consumption.

^2^Model 1+ hypertension, short sleep duration, and trouble sleep.

^3^Model 2+healthy eating index, total calorie intake, tea intake, and physical activity.

IR, insulin resistance; TyG, triglyceride-glucose.

**Table S4.** Association of coffee drinking pattern with insulin resistance indices, stratified by coffee type.

| **Model** | **TyG** | **METS-IR** | **TG/HDL-C** |
| --- | --- | --- | --- |
| ***No suger (black coffee + milk only)*** |  |  |  |
| Non-drinkers | 1 (reference) | 1 (reference) | 1 (reference) |
| All-day type | -0.02(-0.06, 0.03) | -0.35(-0.66,-0.05) | -0.11(-0.41, 0.05) |
| Morning type | **-0.04(-0.07,-0.01)** | **-0.48(-0.7,-0.27)** | **-0.23(-0.41,-0.05)** |
| ***Milk only (no suger)*** | | | |
| Non-drinkers | 1 (reference) | 1 (reference) | 1 (reference) |
| All-day type | -0.01 (-0.06,0.03) | -0.38(-0.68, -0.08) | -0.1(-0.33, 0.12) |
| Morning type | **-0.04(-0.07,-0.01)** | **-0.51(-0.73, -0.28)** | **-0.2(-0.39,-0.02)** |
| ***Black coffee only*** | | | |
| Non-drinkers | 1 (reference) | 1 (reference) | 1 (reference) |
| All-day type | -0.04 (-0.19,0.12) | -0.17(-1.25, 0.91) | -0.24(-1.12, 0.64) |
| Morning type | **-0.07 (-0.14,-0.01)** | -0.36(-0.86, 0.14) | **-0.54(-0.95, -0.14)** |

Data are presented as beta coefficients (β) and 95% confidence interval (CI). Bold font indicates statistical significance (*p* < 0.05). Analyses were performed using weighted generalized linear models (GLMs).

All models were adjusted for age, sex, race and ethnicity, family income, education levels, marry status, coffee intake, smoke status, body mass index, alcohol consumption, hypertension, short sleep duration, and trouble sleep, healthy eating index, total calorie intake, tea intake, and physical activity.

TyG, triglyceride-glucose; METS-IR, metabolic score for insulin resistance; TG, triglyceride; HDL-C, high density lipoprotein_cholesterol.

**Table S5**. **Stratified analyses of** coffee drinking pattern **and METS-IR.**

| Variable | Non-drinker | All-day type | Morning type | *P* for trend | *P* for interaction |
| --- | --- | --- | --- | --- | --- |
| Sex |  |  |  |  | 0.745 |
| Male | 1 (reference) | -0.23(-0.73, 0.26) | **-0.45(-0.84,-0.06)** | **0.02** |  |
| Female | 1 (reference) | **-0.4(-0.79,-0.01)** | **-0.56(-0.82,-0.29)** | **<0.001** |  |
| Age |  |  |  |  | 0.555 |
| ≥45 | 1 (reference) | **-0.6(-1.08,-0.12)** | **-0.62(-0.98,-0.25)** | **0.002** |  |
| <45 | 1 (reference) | -0.2(-0.8, 0.40) | **-0.61(-0.97,-0.24)** | **0.001** |  |
| Education levels |  |  |  |  | 0.301 |
| Less than high school | 1 (reference) | **-0.53(-0.92,-0.14)** | **-0.65(-0.92,-0.37)** | **<0.0001** |  |
| More than high school | 1 (reference) | -0.16(-0.86, 0.55) | **-0.53(-1.01,-0.05)** | **0.027** |  |
| Race |  |  |  |  | **0.017** |
| Non-Hispanic White | 1 (reference) | **-0.88(-1.35,-0.41)** | **-0.9(-1.22,-0.57)** | **<0.001** |  |
| Non-Hispanic Black | 1 (reference) | **-1.33(-2.39,-0.27)** | **-0.74(-1.21,-0.26)** | **0.006** |  |
| Mexican American | 1 (reference) | 0(-0.71,0.71) | -0.08(-0.61,0.45) | 0.747 |  |
| Other Hispanic | 1 (reference) | 0.25(-0.56, 1.05) | -0.37(-0.99, 0.25) | 0.167 |  |
| Sleep duration |  |  |  |  | 0.25 |
| <7 hours | 1 (reference) | 0.12(-0.41 0.65) | **-0.45(-0.81,-0.09)** | **0.004** |  |
| ≥7 hours | 1 (reference) | **-1.17(-2.06,-0.28)** | **-0.95(-1.45,-0.44)** | **<0.001** |  |
| Trouble sleep |  |  |  |  | 0.429 |
| No | 1 (reference) | -0.29(-0.75, 0.18) | **-0.71(-1.02,-0.41)** | **<0.0001** |  |
| Yes | 1 (reference) | **-0.96(-1.8,-0.11)** | **-0.8(-1.44,-0.16)** | **0.027** |  |
| Hypertension |  |  |  |  | 0.366 |
| No | 1 (reference) | -0.35(-0.76, 0.06) | **-0.69(-0.96,-0.42)** | **<0.0001** |  |
| Yes | 1 (reference) | **-0.67(-1.34,-0.01)** | **-0.62(-1.07,-0.16)** | **0.013** |  |
| Total PA |  |  |  |  | 0.341 |
| <150 min/wk | 1 (reference) | **-0.6(-1.06,-0.13)** | **-0.75(-1.1,-0.4)** | **<0.0001** |  |
| ≥150 min/wk | 1 (reference) | -0.36(-0.85, 0.13) | **-0.55(-0.88,-0.22)** | **0.001** |  |

Data are presented as beta coefficients (β) and 95% confidence interval (CI). Bold font indicates statistical significance (*p* < 0.05). Analyses were performed using weighted generalized linear models (GLMs).

All models were adjusted for family income, marry status, alcohol consumption, smoke status, BMI, healthy eating index, total calorie intake, coffee intake, and tea intake.

METS-IR, metabolic score for insulin resistance; PA, physical activity.
